# Correlation between quantitative pivot shift and generalized joint laxity: a prospective multicenter study of ACL ruptures

**DOI:** 10.1007/s00167-017-4785-2

**Published:** 2017-11-17

**Authors:** David Sundemo, Anna Blom, Yuichi Hoshino, Ryosuke Kuroda, Nicola Francesco Lopomo, Stefano Zaffagnini, Volker Musahl, James J. Irrgang, Jón Karlsson, Kristian Samuelsson, The PIVOT Study Group

**Affiliations:** 1 Department of Orthopaedics, Institute of Clinical Sciences, The Sahlgrenska Academy, University of Gothenburg, Gothenburg, Sweden; 2 Närhälsan Torslanda Rehabilitation Center, Torslanda, Sweden; 3 Department of Orthopaedic Surgery, Kobe University, Kobe, Japan; 4 Dipartimento di Ingengeria dell’Informazione, Università degli Studi di Brescia, Brescia, Italy; 5 Laboratorio di Biomeccanica e Innovazione Tecnologica, Istituto Ortopedico Rizzoli, Bologna, Italy; 6 Department of Orthopaedic Surgery, University of Pittsburgh, Pittsburgh, PA USA; 7 Department of Orthopaedics, Sahlgrenska University Hospital, Mölndal, Sweden

**Keywords:** Anterior cruciate ligament reconstruction, Rotatory knee laxity, Pivot shift test, Inertial sensor, Image analysis system, Generalized joint laxity, Generalized joint hypermobility, Beighton Score

## Abstract

**Purpose:**

To investigate whether an increased magnitude of quantitative rotatory knee laxity is associated with a greater level of generalized joint laxity in ACL-injured and contralateral knees.

**Methods:**

A total of 103 patients were enrolled across four international centers to undergo anatomic ACL reconstruction. Rotatory knee laxity was evaluated preoperatively, both in the awake state and under anesthesia, using the standardized pivot shift test. Two devices were used to quantify rotatory knee laxity; an inertial sensor, measuring the joint acceleration, and an image analysis system, measuring the lateral compartment translation of the tibia. The presence of generalized joint laxity was determined using the Beighton Hypermobility Score. The correlation between the level of generalized joint laxity and the magnitude of rotatory knee laxity was calculated for both the involved knee and the non-involved knee. Further, patients were dichotomized into low (0–4) or high (5–9) Beighton Score groups. Alpha was set at < 0.05.

**Results:**

Ninety-six patients had complete datasets, 83 and 13 in the low and high Beighton Score groups respectively. In anesthetized patients, there was a significant correlation between the degree of Beighton Score and quantitative pivot shift when analyzing the non-involved knee using the image analysis system (*r* = 0.235, *p* < 0.05). When analyzing the same knee, multivariate analysis adjusted for meniscal injury, age and gender revealed an increased odds ratio for patients with increased lateral compartment translation to be part of the high Beighton Score group (OR 1.86, 95% CI 1.10–3.17, *p* < 0.05). No other correlation was significant. When analyzing the dichotomized subgroups, no significant correlations could be established.

**Conclusion:**

The findings in this study suggest that there is a weak correlation between generalized joint laxity and the contralateral healthy knee, indicating increased rotatory knee laxity in these patients. Generalized joint laxity does not appear to correlate with rotatory knee laxity in ACL-injured knees.

**Level of evidence:**

Prospective cohort study; level of evidence, 2.

## Introduction

Various risk factors for sustaining an anterior cruciate ligament (ACL) injury have been identified. Important factors include, but are not limited to, knee position and movement at the time of injury, the size of the intercondylar notch, hormonal levels in the bloodstream, gender and the level of generalized joint laxity and knee hyperextension [1–7]. The influence of generalized joint laxity, often measured using the Beighton Hypermobility Score, [8] as a risk factor for ACL injury, was previously the subject of debate [9]. However, recent studies have presented convincing evidence that such a correlation exists [1, 4, 6, 7, 9, 10]. The influence of biomechanical susceptibility in patients with generalized joint laxity might be even higher, since it has been shown that hypermobile individuals tend to adapt their movements, preferring more stable activities [11]. Some researchers recommend patients with generalized joint laxity to participate in preventive physiotherapeutic programs to reduce the risk of ACL injury [4, 6].

It thus appears that generalized joint laxity is associated with the risk of injury to the ACL. However, the reason for the increased relative injury risk in these patients is unknown. One possible contributory cause could be increased knee laxity. Previously, anterior knee laxity has been associated with an increased risk of ACL rupture [4, 7]. Moreover, increased anterior [12, 13] and static rotatory [12, 14] knee laxity has been observed in the contralateral healthy knees of patients with ACL injuries when compared with uninjured control patients, further supporting the theory that increased knee laxity is a risk factor for injury. Currently, it is not known whether ACL injured patients with generalized joint laxity display a greater level of quantitative rotatory knee laxity. Moreover, residual rotary knee laxity post-reconstruction is an undesirable scenario, since a positive pivot shift test has been shown to correlate with symptoms of instability [15, 16] and the risk of osteoarthritis [17]. In theory, if an increase in rotatory knee laxity level could be observed in these patient groups preoperatively, they could be recommended more advanced reconstructive techniques, such as the double-bundle ACL reconstruction or the addition of a lateral extra-articular tenodesis, which have been demonstrated to restore normal knee kinematics more effectively [18–20]. It is therefore of great importance to quantify rotatory knee laxity in patients with generalized joint laxity.

To diagnose ACL injury or to quantify rotatory knee laxity, the pivot shift test is often used [21, 22]. Although the pivot shift test has inherent variability, this can be reduced using a standardized maneuver [23]. Numerous devices have been developed to quantify the pivot shift test objectively [24]. Two devices, recently validated with regard to pivot shift quantification, [25] were utilized in the present study. The devices comprise an inertial sensor, measuring joint acceleration (KiRA, Orthokey, LLC, Lewes, DE, USA), and an image analysis system, measuring the displacement of the lateral compartment, using application software installed on a computer tablet (Apple Inc, Cupertino, CA, USA) [26, 27].

The purpose of this study was to investigate whether (1) ACL-injured knees and (2) healthy contralateral knees demonstrate a higher degree of quantitative rotatory knee laxity, depending on the level of generalized joint laxity. The outcome investigated in the current study, rotatory knee laxity, is defined by measuring joint acceleration and lateral knee compartment translation using the above-mentioned devices. It was hypothesized that patients with generalized joint laxity would display increased rotatory knee laxity, in both the injured and contralateral knees, as interpreted by the technological devices that were used.

## Materials and methods

A prospective observational multi-center study was designed with the aim of examining patients over a period of 24 months. A total of 103 patients were recruited to the study across all sites. Patients were included if they had an injury to one or both bundles of the ACL, were between 14 and 50 years of age, were scheduled for ACL reconstruction within one year of injury and participated regularly (> 100 h) in level I (American football, basketball or soccer) or level II (racquet sports, skiing or manual labor occupations) activities. Patients were excluded if they had grade three or four cartilage lesions in the knee joint, prior ligament surgery on the involved knee, concomitant posterior cruciate ligament injury, an inflammatory arthritic condition, other injury to the lower extremities affecting the ability to walk or participate in level I or II activities or if they had had prior surgery or had a concurrent injury to the contralateral knee. Concomitant meniscal or collateral ligament injuries did not exclude patients from participation. Patients were examined both in the office setting and intraoperatively by arthroscopy to determine whether they were eligible for the study according to the inclusion and exclusion criteria. Sports medicine fellowship-trained orthopedic surgeons performed all the intraoperative examinations and reconstructions between December 2012 and February 2015.

## Baseline evaluation

In order prospectively to observe demographic parameters, baseline evaluations were made preoperatively. Data relating to age, gender, sports activity level and work activity were collected. Determinations of work activity level and sports activity level were made using the Cincinnati Occupational Rating Scale and the Marx Sports Activity Scale respectively [28, 29]. Moreover, additional data relating to patient-reported outcome were collected using the IKDC 2000 and Activities of Daily Living Scale of the Knee Outcome Survey [30, 31]. Baseline data were also collected for clinical tests, such as manual and instrumented measurements of knee joint laxity, goniometric measurements of range of motion and kinematic measurements using two devices for the quantification of the pivot shift test [26, 27].

## Clinical assessments and follow-up

Generalized joint laxity was examined using the original criteria of the Beighton Hypermobility Score presented by Beighton et al. in 1973 [8]. Division into subgroups with high and low Beighton Scores was made for the purpose of analysis. Various cut-off points have been used in the literature [32–35]. Division into 0–4 and 5–9 points was selected for low and high Beighton Score groups respectively, with the aim of only capturing patients with definite hypermobility. The pre-, intra- and postoperative execution of the pivot shift test was performed by sports medicine fellowship-trained orthopedic surgeons on both the injured and the contralateral knee. The maneuver was performed both awake and under general anesthesia in all patients. The same examiner tested each specific patient both awake and under anesthesia. A total of six examiners performed all the pre- and intraoperative tests across all centers. The examiners were not blinded with respect to which knee was examined, either the injured or the non-injured knee. The pivot shift test was performed in a standardized manner to minimize inter-examiner variability [23]. The pivot shift test was first performed manually and quantified in the classical subjective manner according to the IKDC criteria as normal, nearly normal (glide), abnormal (clunk) and severely abnormal (gross) [36].

**Fig. 1:**
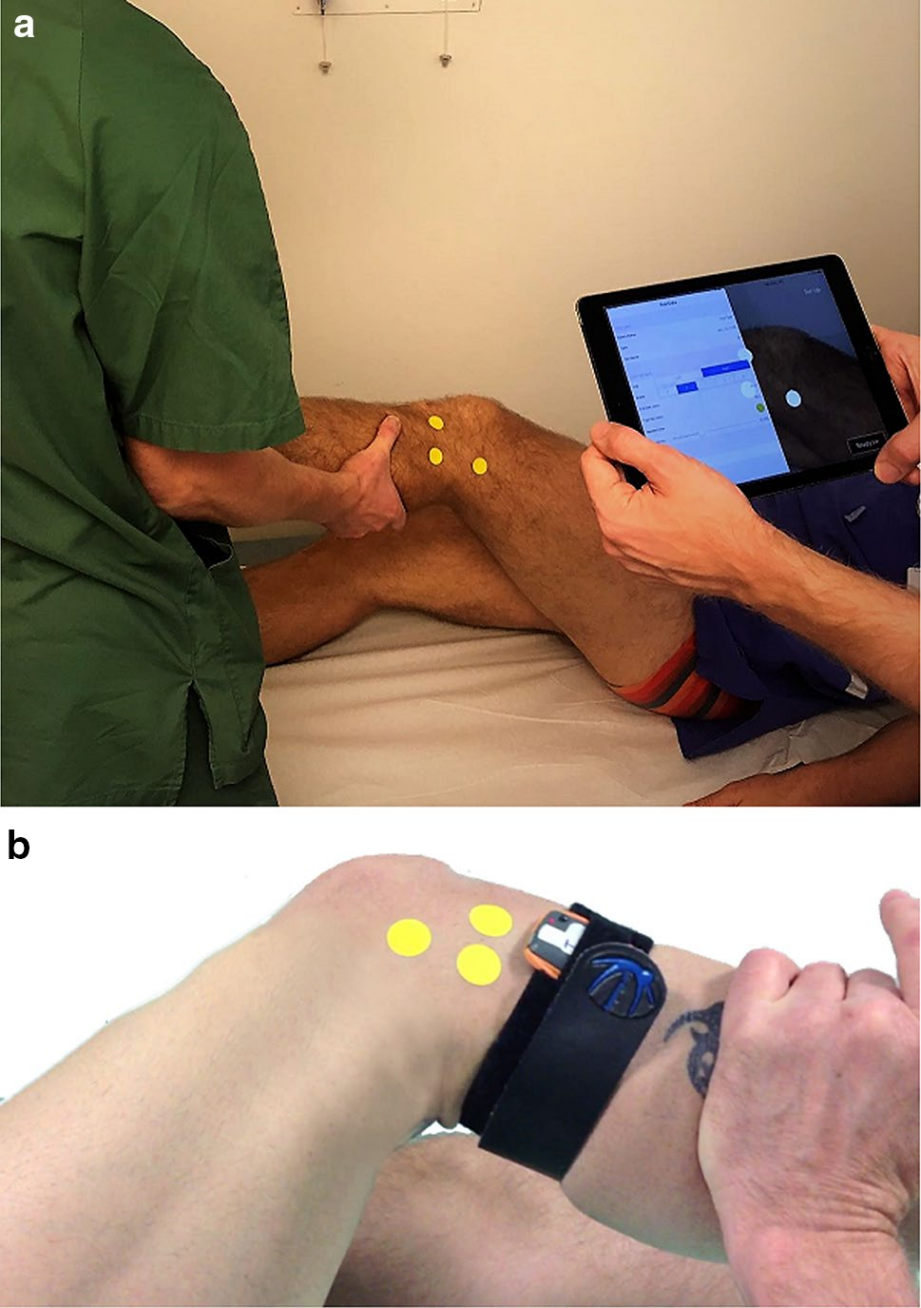
To the above, the setup of the image analysis system is shown (**a**). To the below, the accelerometer, fastened with a Velcro strap, and a close-up of the position of the markers of the image analysis system can be seen (**b**)

**Fig. 2:**
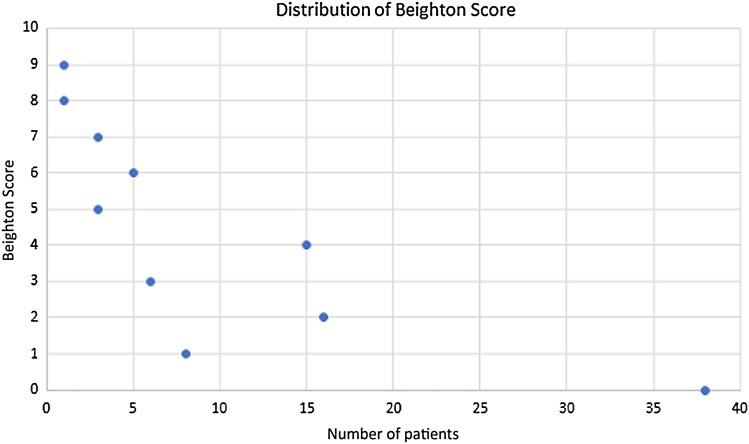
Diagram demonstrating the distribution of Beighton Score for all patients

A device using an inertial sensor was utilized, as recently described by Lopomo et al. [27]. The tri-axial sensor, or accelerometer, was fastened to the lateral aspect of the proximal tibia (Fig. 1a). This device quantifies the pivot shift test by measuring the acceleration of the joint during the execution of the maneuver. It has been tested in terms of reliability, presenting an intra-class correlation coefficient of 0.79. Additionally, a recently developed image analysis system was utilized to measure the translation of the lateral compartment (Figs. 1b, 2) [26]. The lateral aspect of the knee was filmed with a tablet computer using brightly colored markers (Color Coding Labels; Avery Dennison Corporation, Pasadena, CA, USA) applied to three bony landmarks: the tubercle of Gerdy, the lateral epicondyle and the fibular head. The relative two-dimensional movement of the marked structures is captured by the camera and a software program produces a graph plotting the anteroposterior position of the femur as a function of time. The change in position occurring during femoral reduction on the tibia, when the knee reaches its pivot point, is captured and the distance of the shift in position is presented in millimeters [37]. The image analysis system has shown excellent repeatability calculated by measuring intraobserver and interobserver intraclass correlation coefficients (ICCs > 90) [38, 39].

The institutional review board approved the University of Pittsburgh as the coordination center for the study. Moreover, institutional review boards in the individual countries of the three collaborating international centers approved the study (Reference number: 1008-12). All patients gave their written and informed consent prior to inclusion in the study.

## Statistical methods

Correlations between quantitative pivot shift and the level of generalized joint laxity were performed using the Spearman correlation. Analyses of preoperative data presenting descriptive statistics of the two Beighton Score subgroups were made using either the Pearson Chi-square test or the Mann–Whitney *U* test. The means and standard deviations of the descriptive parameters were calculated to show the dispersion of the data. To analyze the outcome data in relation to the subgroups, the Mann–Whitney *U* test was used. A non-parametric Mann–Whitney *U* test was used, since the data were found to be non-normally distributed. Further, the median and interquartile ranges were presented to illustrate the composition of the data. Multivariate analysis of the Beighton Score subgroups was performed using multivariate logistic regression and presented using odds ratios and confidence intervals to illustrate the precision of the estimate. No sample size analysis was made for the purpose of this study. Statistical Alpha was set at 0.05.

## Results

One hundred and three patients were involved in the study. A total of 89 (86%) patients had complete ACL ruptures and the remaining 14 (14%) patients suffered partial ruptures. Data relating to the level of the Beighton Score were missing for seven patients, leading to their exclusion from the analysis. Complete data sets for 96 (93%) patients were analyzed.

Of all the patients included in the analysis, 40 (42%) were female and 56 (58%) were male. The mean age of the whole cohort was 24.6 (± 9.1) years (Table 2). Forty-two percent had a normal or low-grade clinical pivot shift grade (normal or glide), according to the IKDC criteria, whereas 58 percent had a high-grade pivot shift (clunk or gross) (Table 1). As a group, female patients had significantly higher Beighton Scores when compared with male patients (*p* = 0.03). There was a difference showing that the patients in the subgroup with low Beighton Scores were significantly older than the patients in the high Beighton Score group (*p* = 0.047). There was no significant difference between the subgroups of high and low Beighton Scores in terms of the frequency of medial or lateral meniscus involvement (*p* = 0.93, *p* = 0.82), the Cincinnati Occupational Rating Scale (*p* = 0.40), the IKDC 2000 (*p* = 0.55) or the Activities of Daily Living Scale (*p* = 0.42). However, the group with high Beighton Scores had a higher level of activity according to the Marx Activity Scale (*p* = 0.003, Table 2). Quantification using the image analysis system in awake patients did not reveal a significant correlation with the Beighton Score for the involved knee (*p* = 0.837), the non-involved knee (*p* = 0.278) or when measuring side-to-side difference (*p* = 0.744). Further, using the accelerometer in awake patients, no significant correlation could be observed in the involved knee (*p* = 0.475), the non-involved knee (*p* = 0.169) or by measuring the side-to-side difference (*p* = 0.970) (Table 3).

**Table 1:** Preoperative clinical pivot shift grade performed under anesthesia, all patients

	*n* (%)
Pivot shift grade^a^	
Normal	1 (1.0)
Nearly normal	42 (40.8)
Abnormal	52 (50.5)
Severely abnormal	8 (7.8)

^a^According to the International Knee Documentation Committee 2000 form

**Table 2:** Descriptive statistics, baseline data and concomitant meniscal injuries

	All patients	Beighton Score 0–4	Beighton score 5–9	*p* value	*N*
Gender (female/male)	40/56	31/52 (37.3/62.7%)	9/4 (69.2/30.8%)	0.03*	96
Age (mean, ± SD)	24.6 (± 9.1)	25.5 (± 9.5)	18.9 (± 11.8)	0.047*	96
Medial meniscus (normal/lesion)	58/38 (60.4/39.6)	50/33 (60.2/39.8%)	8/5 (61.5/38.5%)	0.93 (n.s.)	96
Lateral meniscus (normal/lesion)	58/38 (60.4/39.6)	53/30 (63.9/36.1%)	5/8 (38.5/61.5%)	0.82 (n.s.)	96
CORS (mean, ± SD)	29.4 (± 14.9)	29.2 (± 15.3)	30.6 (± 11.8)	0.40 (n.s.)	96
MAS (mean, ± SD)	11.3 (± 5.2)	10.7 (± 5.4)	14.9 (± 2.2)	0.003*	96
IKDC 2000 (mean, ± SD)	58.1 (± 15.7)	58.4 (± 15.7)	55.8 (± 16.2)	0.55 (n.s.)	96
ADLS (mean, ± SD)	79.8 (± 15.7)	80.3 (± 15.7)	76.7 (16.4)	0.42 (n.s.)	96

*CORS* Cincinnati Occupational Rating Scale, *MAS* Marx Activity Scale, *IKDC* International Knee Documentation Committee, *ADLS* Activities of Daily Living Scale of the Knee Outcome Survey, *SD* Standard deviation

*Statistical significance, *p* values are provided for analysis of the difference between the Beighton Score subgroups, *n.s*. non-significant

**Table 3:** Correlation with Beighton Score, preoperative awake patients

	Correlation (rho)	*p* value	*N*
IAS—involved knee	− 0.022	0.837 (n.s.)	92
IAS—non-involved knee	− 0.117	0.278 (n.s.)	88
IAS—side-to-side difference	0.035	0.744 (n.s.)	88
Accelerometer—involved knee	− 0.079	0.475 (n.s.)	84
Accelerometer—non-involved knee	− 0.154	0.169 (n.s.)	82
Accelerometer—side-to-side difference	− 0.004	0.970 (n.s.)	82

*IAS* image analysis system, *n.s*. non-significant

*Statistical significance

In anesthetized patients, there was no evident correlation between the quantitative data obtained using the image analysis system and the level of Beighton Score when analyzing the involved knee (*p* = 0.309) or the side-to-side difference (*p* = 0.762). However, when analyzing the non-involved side, there was a significant correlation (*r* = 0.235, *p* = 0.024). Using the accelerometer, no significant correlations could be established with patients under anesthesia (Table 4). Using the Mann–Whitney *U* test to compare subgroups with high and low Beighton Scores, there was no significant difference either when using the image analysis system or when using the accelerometer when examining the involved knee, the non-involved knee or the side-to-side difference. The results did not reach statistical significance either when patients were awake or when they were under anesthesia (Tables 5, 6). Multivariate analysis, adjusted for meniscal injuries, age and gender, showed that the image analysis system was able successfully to predict the level of Beighton Score in the non-involved knee in anesthetized patients. No other analysis, using multivariate statistics, was significant (Table 7).

**Table 4:** Correlation with Beighton Score, preoperative anesthetized patients

	Correlation (rho)	*p* value	*N*
IAS—involved knee	0.106	0.309 (n.s.)	95
IAS—non-involved knee	0.235*	0.024*	92
IAS—side-to-side difference	− 0.032	0.762 (n.s.)	92
Accelerometer—involved knee	0.138	0.217 (n.s.)	82
Accelerometer—non-involved knee	0.027	0.812 (n.s.)	82
Accelerometer—side-to-side difference	0.125	0.265 (n.s.)	82

*IAS* image analysis system, *n.s*. non-significant

*Statistical significance

**Table 5:** Beighton Score dichotomized, preoperative awake patients

	Beighton Score 0–4 (*n*, median: IQR)	Beighton Score 5–9 (*n*, median: IQR)	*p* value
IAS—involved knee	77, 2.00: 1.76	12, 1.65: 0.53	0.322 (n.s.)
IAS—non-involved knee	75, 0.80: 0.80	12, 0.52: 1.15	0.416 (n.s.)
IAS—side-to-side difference	76, 1.00: 0.80	12, 0.83: 1.52	0.576 (n.s.)
Accelerometer—involved knee	71, 3.50: 2.40	13, 3.50: 2.90	0.809 (n.s.)
Accelerometer—non-involved knee	69, 2.70: 1.35	13, 2.60: 1.45	0.985 (n.s.)
Accelerometer—side-to-side difference	69, 0.60: 1.55	13, 0.60: 1.70	0.785 (n.s.)

*IAS* image analysis system, *SD* standard deviation, *IQR* interquartile range, *n.s*. non-significant

*Statistical significance

**Table 6:** Beighton Score dichotomized, preoperative anesthetized patients

	Beighton Score 0–4 (*n*, median: IQR)	Beighton Score 5–9 (*n*, median: IQR)	*p* value
IAS—involved knee	82, 2.65: 2.61	13, 2.80: 3.24	0.721 (n.s.)
IAS—non-involved knee	79, 0.70: 0.90	13, 1.00: 2.17	0.149 (n.s.)
IAS—side-to-side difference	79, 1.70: 2.34	13, 1.24: 1.34	0.260 (n.s.)
Accelerometer—involved knee	69, 4.40: 2.75	13, 4.90: 6.60	0.310 (n.s.)
Accelerometer—non-involved knee	69, 2.70: 1.20	13, 3.00: 1.20	0.263 (n.s.)
Accelerometer—side-to-side difference	69, 1.80: 2.50	13, 1.60: 5.75	0.939 (n.s.)

*IAS* image analysis system, *SD* standard deviation, *IQR* interquartile range, *n.s*. non-significant

*Statistical significance

**Table 7:** Beighton score, multivariate analysis of preoperative anesthetized patients

	OR	OR CI (95%)	*p* value
IAS—involved knee	1.07	0.81–1.41	0.650 (n.s.)
IAS—non-involved knee	1.86	1.10–3.17	0.022*
IAS—side-to-side difference	0.85	0.60–1.19	0.333 (n.s.)
Accelerometer—involved knee	1.06	0.93–1.19	0.387 (n.s.)
Accelerometer—non-involved knee	1.40	0.84–2.32	0.196 (n.s.)
Accelerometer—side-to-side difference	1.04	0.92–1.16	0.556 (n.s.)

*IAS* image analysis system, *OR* odds ratio, *CI* confidence interval, *n.s*. non-significant

*Statistical significance. Multivariate analysis adjusted for meniscal injuries, age and gender

## Discussion

The main finding in this multi-center study was the non-existent correlation between the degree of generalized joint laxity and assessed quantitative pivot shift measurements in knees with an ACL injury. This is the first study to assess this issue and it might be too early to make conclusive statements. However, there is currently no reason to recommend different treatment regimens for ACL reconstruction in patients with generalized joint laxity based on preoperative laxity measurements alone. Moreover, the results of this study indicate that it is perhaps more likely that a possible difference in knee kinematics between healthy and hypermobile patients is primarily due to differences in the structure and function of the anterior cruciate ligament itself. This theory is founded on the fact that there was a slight, yet apparent, correlation in the contralateral healthy knee in anesthetized patients. To elaborate, the traumatic knee injury causing ACL rupture will significantly affect the rotational knee laxity measurements [40, 41]. Intact knees would, therefore, in theory, better correspond to the innate level of generalized joint laxity than injured knees.

A significant correlation was found between rotatory knee laxity in healthy knees and the level of generalized joint laxity using the Spearman correlation analysis. To validate the results, we performed multivariate analysis to adjust for potential confounders. Meniscal injuries are known to affect the degree of rotatory knee laxity [41, 42]. Further, age and gender are factors that influence the amount of joint laxity [10, 43, 44]. Adjusting for these three factors, we were able to consolidate the previously established connection between the image analysis system and generalized joint laxity in the non-involved knee. This analysis demonstrates that a greater translation of the lateral compartment is associated with a greater likelihood of having generalized joint laxity. Although both the Spearman correlation and multivariate analysis were significant, the correlation was weak and further studies are warranted to verify these results. The inclusion of healthy control patients in future studies would increase the quality of evidence. With this in mind, this finding implies the possible existence of a link between generalized joint laxity and the magnitude of rotatory knee laxity in healthy knees.

The correlation identified in the non-involved knee using the image analysis system could not be verified using the inertial sensor. This could possibly be ascribed to the fact that acceleration is more sensitive to the overall dynamics reached by the joint during the maneuver, especially during the phase of reduction. Generalized joint laxity could be hypothesized to influence the overall range of rotation, and correspondingly, to have a greater effect on the displacement of the lateral compartment.

Furthermore, a correlation could not be seen in the awake state, perhaps owing to the factor of muscular guarding complicating the interpretation of the pivot shift test in awake patients. Interestingly, previous studies have shown that the determination of rotatory knee laxity is more correct when patients are under general anesthesia when muscular guarding is not an issue. To exemplify, Nakamura et al. evaluated rotatory knee laxity using the same inertial sensor used in this study by examining patients both in the awake state and under general anesthesia [45]. Using the pivot shift test, no difference between ACL-injured and ACL-intact knees could be observed when patients were awake. On the other hand, when patients were under anesthesia, there was a significant difference in posterior tibial acceleration [45]. Similarly, Lopomo et al. found significant differences in acceleration and anteroposterior translation when comparing awake and anesthetized patients [46]. Consequently, it is fair to assume that examinations performed under general anesthesia are more precise in the determination of rotatory knee laxity in patients.

Generalized joint laxity is regarded as a risk factor for ACL injury, but little is known about its influence on rotatory knee laxity. Interest has recently focused on the implications of quantitative rotatory knee laxity using the pivot shift test [26, 27, 47–53]. Since a pathological pivot shift test correlates with an inferior clinical outcome and the development of osteoarthritis, [16, 17, 54] it is important to quantify rotatory knee laxity. This is particularly important in groups of patients susceptible to sustaining ACL injury and ACL re-injury, since improved knowledge could facilitate the development and implementation of prophylactic exercises, a recommendation which has already been made in the literature [4, 6]. An improved knowledge of knee kinematics in patients with generalized joint laxity is important further to understand why these patients run an elevated risk of sustaining knee injuries.

There are a few limitations to this study. First, the interpretation of the pivot shift test is difficult and both instrumented and manual quantification may vary between examiners [55]. This issue was mitigated by the implementation of the standardized pivot shift test, which has been shown to minimize variability [23]. Second, a skewing of the descriptive data between the subgroups with a high or low Beighton Score could be observed for gender, age and Marx Activity Scale. It has been shown that female gender and younger age correlate with a higher level of generalized joint laxity [10, 43, 44]. This was verified by the present study. It could therefore be hypothesized that the skewing in terms of age and gender was not coincidental but rather causal or at least probable, owing to the increased risk of hypermobility in these subgroups. Since the unbalanced groups can be regarded as natural, this should not interfere with the analysis. Further, to adjust for the unbalanced data, multivariate analysis was performed. The difference in Marx Activity Scale is more difficult to explain. A recent study indicates that patients with generalized joint laxity tend to avoid more unstable activities, [11] a fact that was contradicted by the present study. Due to the relatively small subgroups with high Beighton Scores, this might be a coincidental finding and a potential bias in the analysis of the data. It could be hypothesized that patients in this particular study with a high Beighton Score and a high Marx Activity Rating scale might have unrepresentatively stable knee joints for their particular level of Beighton Score, which could bias the results.

## Conclusion

In conclusion, the findings in this study suggest a weak correlation between generalized joint laxity and the contralateral healthy knee, indicating increased rotatory knee laxity in these patients. Generalized joint laxity does not appear to correlate with rotatory knee laxity in ACL-injured knees.
